# Development of Sedative Dexmedetomidine Sublingual In Situ Gels: In Vitro and In Vivo Evaluations

**DOI:** 10.3390/pharmaceutics14020220

**Published:** 2022-01-18

**Authors:** Ayat A. Allam, Nermin E. Eleraky, Nadeen H. Diab, Mahmoud Elsabahy, Sahar A. Mohamed, Hala S. Abdel-Ghaffar, Nivin A. Hassan, Samia A. Shouman, Mervat M. Omran, Sahar B. Hassan, Noura G. Eissa

**Affiliations:** 1 Pharmaceutics Department, Faculty of Pharmacy, Assiut University, Assiut 71526, Egypt; ayat.alam@pharm.aun.edu.eg (A.A.A.); nermineleraky@pharm.aun.edu.eg (N.E.E.); 2 Pharmaceutics Department, Faculty of Pharmacy, Sphinx University, Assiut 71515, Egypt; 3 School of Biotechnology, Badr University in Cairo, Badr City 11829, Egypt; nadeendiab37@gmail.com; 4 Department of Chemistry, Texas A&M University, College Station, TX 77842, USA; 5 Anesthesia, Intensive Care and Pain Management Department, South Egypt Cancer Institute, Assiut University, Assiut 71111, Egypt; drsaher2008@aun.edu.eg; 6 Anesthesia and Intensive Care Department, Faculty of Medicine, Assiut University, Assiut 71111, Egypt; hala_abdelghaffar@med.aun.edu.eg; 7 Pharmacology and Experimental Oncology Unit, Cancer Biology Department, South Egypt Cancer Institute, Assiut University, Assiut 71111, Egypt; nivin2@aun.edu.eg; 8 Pharmacology and Experimental Oncology Unit, Cancer Biology Department, National Cancer Institute, Cairo University, Cairo 11796, Egypt; samia.shouman@nci.cu.edu.eg (S.A.S.); Mervat.omran@nci.cu.edu.eg (M.M.O.); 9 Clinical Pharmacy Department, Faculty of Pharmacy, Assiut University, Assiut 71526, Egypt; sahar.badr@pharm.aun.edu.eg; 10 Department of Pharmaceutics and Industrial Pharmacy, Faculty of Pharmacy, Zagazig University, Zagazig 44519, Egypt; nouraeissa@zu.edu.eg

**Keywords:** dexmedetomidine, selective α-2 adrenergic agonist, sublingual, in situ gels, pharmacokinetics, pharmacodynamics

## Abstract

Intravenous dexmedetomidine (DEX) is currently approved by the FDA for the sedation of intubated patients in intensive care units to reduce anxiety and to augment postoperative analgesia. Bradycardia and hypotension are limitations associated with the intravenous administration of DEX. In this study, DEX sublingual in situ gels were developed and assessed for their pH, gelling capacity, viscosity, mucoadhesion and in vitro drug release. The optimized gelling system demonstrated enhanced mucoadhesion, superior gelling capacity, reasonable pH and optimal rheological profile. In vivo, compared to the oral solution, the optimal sublingual gel resulted in a significant higher rate and extent of bioavailability. Although the in situ gel had comparable plasma levels to those observed following intravenous administration, significant amelioration of the systemic adverse reactions were attained. As demonstrated by the hot plate method, a sustained duration of analgesia in rats was observed after sublingual administration of DEX gel compared to the intravenously administered DEX solution. Furthermore, no changes in systolic blood pressure and heart rate were recorded in rats and rabbits, respectively, after sublingual administration of DEX. Sublingual administration of DEX in situ gel provides a promising approach for analgesia and sedation, while circumventing the reported adverse reactions associated with intravenous administration of DEX.

## 1. Introduction

Dexmedetomidine (DEX) is an α-2 adrenoceptor agonist with high selectivity that possesses sedative, sympatholytic, anxiolytic and analgesic effects with minimal effects on the respiratory and gastrointestinal systems. The sympatholytic effect of DEX reduces stress response during surgeries and perioperative period. For all the aforementioned favorable physiological effects, DEX is currently approved by the FDA for intravenous (IV) bolus administration and as an infusion for sedation of intubated patients in intensive care units and in procedural sedation during surgical operations to reduce anxiety and to augment postoperative analgesia [1,2]. However, IV administration of DEX is mainly limited by systemic adverse effects such as bradycardia and hypotension.

Research endeavors by our group and others focused on drug delivery approaches that could offer improved stability, bioavailability and therapeutic outcomes compared to the commercially available medications [3,4,5]. Currently available DEX formulations (Precedex^®^, Dexdor^®^) are prescribed for IV administration. Due to the aforementioned limitations of IV DEX, in situ forming gel was proposed in the current study for sublingual administration of DEX to induce sedation and analgesia while controlling over the fluctuations in heart rate and blood pressure. In situ gels are solutions that form gels upon exposure to physicochemical changes in the medium (e.g., change in pH or temperature) [6]. Gels degrade slowly while allowing the release of the entrapped drugs over an extended period of time. Administration of gel prolongs the contact time of the entrapped drug onto mucosa, thus increased absorption and higher bioavailability are usually achieved [7,8]. Our group have exploited in situ gelling systems previously for the delivery of various drugs (e.g., betaxolol and vancomycin) for ocular administration [3,9]. Sublingual and buccal administration of sedative and anxiolytic agents have not been widely explored. Oral mucosal drug delivery is a simple non-invasive approach for improving systemic drug absorption while bypassing the hepatic first pass metabolism [10,11,12]. Sublingual route of administration results in a quick absorption and onset of action due to the high vascularization of the oral mucosa [13].

We have previously examined the clinical efficacy of sedative premedication of DEX in situ gel for postoperative analgesic effect for women with breast cancer undergoing radical mastectomy, and the gels have demonstrated practical, effective and safe means of sedation in adults [14]. The focus of the current study was to explore the physical-chemical and biopharmaceutical characteristics of DEX in situ gels, as well as, to investigate in vivo pharmacokinetics and pharmacodynamics of the developed system in animal models, and further to evaluate their ability to ameliorate the systemic side effects associated with the intravenous administration of the free DEX solution.

## 2. Materials and Methods

### 2.1. Materials

Dexmedetomidine (DEX) was purchased from Elmehy engineering Co., (USP1179333, Cairo, Egypt). Hydroxyl ethyl cellulose (HEC), Carbopol 934 (CP 934P) and sodium alginate of medium viscosity (3500 cps for a 2% solution at 25 °C) were purchased from Dow Chemical Co., (Midland, MI, USA). Benzalkonium chloride, sodium hydroxide, potassium phosphate monobasic, sodium phosphate dibasic and boric acid were purchased from Sigma-Aldrich Co., (St. Louis, MO, USA). Porcine stomach mucin (type II), methanol and acetonitrile were purchased from Sigma-Aldrich Co., Chitosan of medium molecular weight was purchased from Fluka Chemie AG (Buchs, Switzerland). All chemicals and reagents were of analytical grades and were used as received without further purification steps.

### 2.2. Preparation of In Situ Gelling Systems

Aqueous solutions of different pH-sensitive polymers (i.e., carbopol 934P (CP 934P), chitosan and sodium alginate (Na alginate)), and hydroxy ethyl cellulose (HEC), as a viscosity enhancing polymer, were prepared as described earlier [3]. Briefly, in situ gel-forming system, pH-triggered type, was prepared by adding HEC (0.2% *w*/*v*) to 75 mL MilliQ water that contains boric acid as an isotonic modifier and benzalkonium chloride as a preservative. Varying concentrations of the used polymer, CP 934P, chitosan or Na alginate were then added to hydrate overnight (Supplementary Materials, Table S1). The pH was adjusted by using 0.5 M NaOH, followed by constant stirring, and the volume was completed to 100 mL using MilliQ water. For medicated gels, DEX (500 µg) was dissolved in water before the addition of the gel-forming polymers. The physical state and general appearance (i.e., color and clarity) for each formulation were then evaluated by visual inspection under both physiological and non-physiological conditions. Formulations that possessed the desired rapid sol-to-gel transition at physiological environment were selected for further processing. The prepared gels were sterilized by autoclaving (121 °C, 15 psi, 20 min).

### 2.3. Evaluation of the Formulated In Situ GELLING Systems

#### 2.3.1. Physicochemical Characterizations

The developed in situ gels were evaluated for general appearance, pH, gelling capacity and viscosity. A pH meter (Mettler Toledo, Greifensee, Switzerland) was used for measuring the pH of the prepared formulations. For the determination of gelling capacity, one drop of the formulation was placed in a 5 mL vial containing 2 mL freshly prepared artificial salivary fluid, ASF (pH 6.8) composed of sodium chloride (8 g/L), potassium phosphate monobasic (0.19 g/L) and sodium phosphate dibasic (2.38 g/L) equilibrated at 37 °C. Visual assessment of gel formation was performed, and the time of gelation and the time required for the formed gel to dissolve were reported [15]. All the viscosity measurements of the prepared gels were performed at selected fixed rotation velocity (5 rpm) using a Brookfield digital DV-III Model viscometer (Brookfield Engineering Laboratories, Inc., Stoughton, MA, USA) using its small volume adaptor with a spindle 00.

#### 2.3.2. Mucoadhesion

The prepared in situ gels were evaluated for their mucoadhesion following a previously described method [9]. The prepared in situ gelling solution was warmed at 34 °C while stirring prior to mixing with warmed (34 °C) previously prepared mucin (type II) dispersion in citrate buffer (15% *w*/*v*, pH 4.5). The viscosities of the in situ gel before and after mixing with mucin dispersion and that of the mucin dispersion alone were determined using the Brookfield digital DV-III Model viscometer (spindle 96 of the gel at fixed 10 rpm). The mucoadhesion was then calculated as reported previously [9], using Equations (1) and (2).

(1) $${ƞ}^{b}={ƞ}^{t}-\left({ƞ}^{m}+{ƞ}^{p}\right)$$

(2) $$F^{b}={ƞ}^{b}*\;\gamma$$

where ƞ^b^ is the viscosity increase due to mucoadhesion, ƞ^t^ is the viscosity of the mixture, ƞ^m^ is the viscosity of mucin, ƞ^p^ is the viscosity of the in situ gelling solution, the mucoadhesive force (*F*^b^) and γ represents the shear rate at which the viscosity value was determined.

#### 2.3.3. Rheological Examination

The viscosity and elastic properties of the prepared in situ gels were assessed by evaluating their rheological behaviors. For this, the developed formulation was poured into the small sample adaptor of the Brookfield digital DV-III Model viscometer utilizing a spindle 00, and the rotational speed was gradually increased from 5 to 75 rpm at room temperature. The formulation was then transferred to a semi-solid jar and converted to gel by raising the pH to 6.8 using 0.5 M NaOH. The spindle 96 of the viscometer under the same shear rates was then used to evaluate the rheology of the resultant gel. The corresponding rheograms were built from the calculated mean values of each experiment performed in triplicates.

#### 2.3.4. In Vitro Drug Release

The release of DEX from the selected medicated in situ gels was performed as previously reported with slight modifications [4]. Briefly, the tested formulation (equivalent to 100 µg DEX) was placed in beakers, each containing 20 mL of simulated salivary fluid (pH 6.8) at 37 °C and 100 rpm. After centrifugation of the withdrawn samples (1 mL) at specified time intervals, the filtered supernatant was assayed using a specific and sensitive gas chromatographic mass spectrometric (GC–MS) method (AB SCIEX 3200 Q TRAP, Darmstadt, Germany) equipped with electrospray ionization (ESI) source and an Agilent 1260 affinity HPLC system, consisting of a vacuum degasser, a binary pump and an autosampler [16]. Sink conditions were maintained by replacing each withdrawn sample with equal volume of fresh simulated salivary fluid. In vitro release studies were conducted in triplicates and the mean cumulative drug released was constructed as a function of time. The kinetics of drug release from the studied in situ gels were evaluated as reported previously [17,18].

### 2.4. Stability Study

The stability study of the selected DEX in situ gel (F3) was performed after 30- and 60-days storage periods at three selected different temperatures, 4 °C, room temperature and 40 °C. For this, different samples of the selected gel were prepared and firmly packed in sealed autoclavable transparent glass vials. At each time point, samples from each temperature were evaluated for their surface pH, viscosity, mucoadhesion and gelling capacity. The stability study was constructed at triplicate.

### 2.5. In Vivo Pharmacokinetics Studies

The protocol was approved by the Institutional Animal Ethical Committee of Faculty of Pharmacy, Assiut University (protocol code S18-21 and 7 September 2021 of approval), and it adheres to the Guide for the Care and Use of Laboratory Animals, 8th Edition, National Academies Press, Washington, DC, USA.

The plasma concentrations of DEX were measured in healthy rabbits (3 groups, 5 animals each) after sublingual administration of the selected in situ gel (F3), oral and IV free drug solutions (equivalent to 1 µg/kg DEX). Fifteen healthy rabbits (1.5–2.25 kg) were anesthetized with 0.1 mL thiopentane (0.5 mg/mL). The first group received the selected in situ gel (F3) sublingually, gastric intubation of oral drug solution was performed to the second group, and a single 5 min infusion of 0.2 µg/kg/min as a bolus dose of drug solution (IV bolus) was administered to the third group. Withdrawn blood samples (2 mL) prior to administration (control), and at 15, 30, 45, 60, 120, 180, 240, 300 and 360 min, following drug administration, were collected in heparinized tubes and then centrifuged at 2500× *g* for 10 min for separating the plasma that was stored frozen at −20 °C.

For measuring the plasma concentrations of DEX, a previously reported method [14] was utilized using a specific and sensitive gas chromatographic mass spectrometry (GC–MS, AB SCIEX 3200 Q TRAP, Darmstadt, Germany) equipped with electrospray ionization source and an Agilent 1260 affinity HPLC system, consisting of a vacuum degasser, a binary pump and an autosampler [16]. In total, a 1 mL solvent mixture (methanol: acetonitrile, 1:1, *v*/*v*) was mixed with 500 µL of plasma, and centrifuged for 15 min (10,000× *g*) at 4 °C. The supernatant (10 µL) was injected into LC/MS/MS system (AB SCIEX 3200 Q TRAP, Germany) equipped with electrospray ionization source and an Agilent 1260 affinity HPLC system. Data acquisition and processing were assessed using Analyst 1.6 software. Analysis was performed using XBridge-C18 analytical column (150 mm × 2.1 mm × 5 µm, Waters) at 25 °C. Two solvents were used for the preparation of the mobile phase, solvent A (0.1% formic acid in water) and solvent B (methanol and acetonitrile (1:1, *v*/*v*)). A 0.3 mL/min flow rate was set for running. Serial dilutions of standards were prepared at concentrations that range from 50–1000 ng/mL in plasma and simulated salivary fluid. DEX was detected at a retention time of 3.56 min (in salivary fluids) and 4.4 min (in plasma). Quantification for DEX was performed with multiple reactions monitoring (MRM) by using previously reported ion transitions, declustering potential and the collision energy [14].

DEX pharmacokinetic parameters were determined by fitting the plasma drug concentration time profile to the most correlated model, using Win Nonlin 8.3 Phoenix 64^®^ software (Certara^©^, Princeton, NJ, USA, Inc.). By applying a non-compartmental method, parameters including the area under the plasma DEX concentration versus time curve (AUC), elimination rate constant (Kel) and half-life (t_½_), apparent volume of distribution (V/f) and apparent clearance (CL/f) were calculated. Further, maximum plasma concentration (C_max_), time to reach C_max_ (t_max_), were determined. DEX absolute bioavailability after extravascular administration was calculated by comparing the corresponding extravascular AUC to that after intravenous bolus route.

### 2.6. Pharmacodynamics Studies

Hot plate test and blood pressure measurements were performed in rats that were randomly divided into four groups (6 animals each). The first group served as the control group (received no treatment), the second group received a single dose of oral DEX solution (1 µg/kg) via oral gavage, the third group received IV DEX as a single 5 min infusion of 0.2 µg/kg/min (bolus dose), while the fourth group received sublingual DEX in situ gel (F3). Heart rate measurements were performed in rabbits that were randomly divided into four groups (5 animals each) as previously described.

#### 2.6.1. Hot Plate Test

The hot plate experiment was conducted as previously reported [19]. Briefly, rats were placed gently onto a 50 °C hot plate. The latency time observed for each rat to exhibit nociceptive responses (e.g., hind leg flinching, paw licking and jumping) was determined at 10, 20, 30, 45, 60 and 120 min after drug administration.

#### 2.6.2. Cardiovascular Effects

##### Measurement of Blood Pressure

Blood pressure measurements were performed using a previously reported tail-cuff method [20] using male Wistar rats (180–220 g). Prior to DEX administration, repeated initial measurement of mean systolic blood pressure was carried out. Following drug administration, systolic blood pressure was then measured after different time intervals (10, 20, 30, 45, 60 and 120 min) using appropriate tail-cuff (LE5001, PanLab™, Harvard Apparatus, Barcelona, Spain) by attaching a cuff with a pneumatic pulse sensor to the tail of each rat. At least three consecutive readings were performed for calculating the average mean systolic blood pressure for each rat at each time point. Rats were trained for this procedure daily for at least three previous days before measurement [20].

##### Measurement of Heart Rate

For measuring the mean heart rate, the surface manifestation of the repolarization and depolarization waves of the heart were represented through electrocardiograph (ECG) pictures utilized according to a previously reported method [21]. During the experiments, anesthetized New Zealand rabbits (1.8–2.0 kg) with intraperitoneal injection of urethane solution (25% *v*/*v*) were canulated with tracheal polyethylene tube. A cannulated right jugular vein was used for IV administration. Animals were ventilated normally with keeping the body temperature at 37 °C. Subcutaneously, the Cardisuny needles were fixed for measuring the pulse rate prior to DEX administration (control), and at 10, 20, 30, 45, 60 and 120 min after drug administration. At least three consecutive readings were performed for calculating the average heart rate for each rabbit at each time point.

### 2.7. Statistical Analysis

Statistical analyses were performed by one-way ANOVA or two-sided Student’s *t*-test for pairwise comparison (GraphPad Prism 6.0, GraphPad, San Diego, CA, USA). Differences between means were considered statistically non-significant (NS) if the *p* value was > 0.05, significant for 0.05 > *p* ≥ 0.01, and highly significant for 0.01 > *p* ≥ 0.001.

## 3. Results and Discussion

### 3.1. Formulation and Characterization of In Situ Gelling System for Sublingual Administration of DEX

Our group has developed in situ gelling systems for the delivery of different drugs for various biomedical applications [3,9]. The current study is aimed to prepare DEX in situ gel that is suitable for sublingual application. Once administered, the polymeric solution should undergo instantaneous transition into a gel that maintains consistency to allow for an improved absorption and bioavailability of DEX. The pH-triggered in situ gel-forming systems were prepared using various concentrations of the polymers (i.e., CP, chitosan, and Na alginate) in combination with HEC, a viscosity-enhancing agent. CP is commonly used in the preparation of in situ gels owing to its sol–gel transition properties in aqueous solution at pH values >5.5 [22]. In situ niosomal gel composed of CP and hydroxypropyl methyl cellulose enhanced the biocompatibility and antibacterial activity of vancomycin upon ocular administration [9]. Furthermore, mucoadhesive CP 934 in situ nanogel was successfully used for the buccal delivery of celecoxib [23]. Chitosan, a cationic biodegradable polymer, is a pH-sensitive polymer (i.e., exhibits a sol–gel transition when the media changes from slightly acidic to neutral (at a pH value of 6.5)) [22]. Additionally, chitosan possesses mucoadhesive and permeation enhancement properties. Several studies have demonstrated the ability of chitosan to form in situ gels for a diversity of biomedical applications [24,25,26]. Na alginate is a natural gel-forming mucoadhesive polymer that is used in the formation of in situ gels especially in combination with other polymers for enhanced mechanical stability [22,27]. In the present study, different in situ gelling systems were prepared using various concentrations of CP 934, chitosan, and Na alginate in the presence of HEC (Supplementary Materials, Table S1). Formulations that were in a liquid form at non-physiological conditions (pH 5) and demonstrated a rapid sol–gel transition at physiological pH 7.4, were selected for further characterizations (Supplementary Materials, Table S1).

The ability of in situ gels to maintain their integrity after administration is crucial for the proper delivery of drugs. Thus, the developed gels were evaluated for their gelling capacity, pH, viscosity and mucoadhesion. The gelling capacity determines the time duration for the gel to remain intact upon contact with artificial salivary fluid before being dissolved. As shown in Table 1, F6, F10 and F11 formed gels that dissolve rapidly, while F2, F7 and F12 showed immediate gelation that remain for few minutes. F3 composed of 0.3% *w*/*v* CP 934 showed the highest gelling capacity (i.e., gave immediate gelation while remained intact for an extended period >1.5 h). The pH values of the prepared in situ gels were in the range of 4.9 ± 0.2 and 6.5 ± 0.2 (Table 1), which were suitable for sublingual administration. The viscosity of the gels ranged from 17.5 ± 3.02 to 37.46 ± 0.82 Pa S. F3 showed the highest viscosity that could be attributed to the high gelling capacity of the polymer used (i.e., CP 934) and the enhanced crosslinking of the polymer at higher concentration (0.3% *w*/*v*) which increases when the pH of the medium increases from acidic to neutral [24,28].

Mucoadhesion is a potential parameter for in situ formulations to maintain a strong interaction with the mucosal surface to prevent rapid drainage and allow for an enhanced drug transport, and thus, reduce frequency of administration [9]. In situ gels composed of CP 934 or chitosan showed higher mucoadhesive properties compared to gels formed using Na alginate, with superior mucoadhesive force observed in case of F3 (Table 1). This could be attributed to the presence of numerous hydrophilic amino, hydroxyl and carboxylates groups in both chitosan and CP 934. These hydrophilic groups enable hydrogen bonding and electrostatic interactions between the polymers and the oral mucosa [9,22,26]. Moreover, the presence of hydrophilic groups exposes the maximum number of adhesive sites through enhancing the polymer swelling in water.

**Figure 1:**
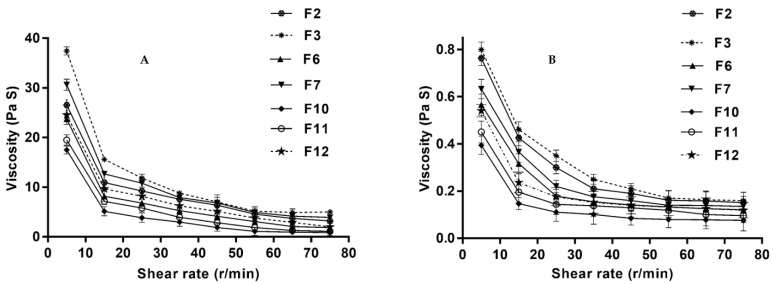
Rheological profiles of DEX in situ gel formulations at physiological (**A**) and non-physiological conditions (**B**).

Under low shear rates, ideal viscoelastic fluids should exhibit high viscosity while exhibiting low viscosity at high shear rates [29]. The rheological behaviors of the prepared formulations were determined by measuring the viscosity of the gels at both physiological (pH 7.4) and non-physiological (pH 5) conditions. The viscosity of the prepared gels decreased as the shear rate increased, thus showing a pseudoplastic behavior (Figure 1A). The selected formulations are suitable for sublingual administration where the shear rate is expected to increase upon administration. In situ gelling systems at non-physiological conditions (i.e., at pH 5) were in the form of solutions (i.e., low viscosity) that underwent slight changes upon increasing the shear rate (Figure 1B).

### 3.2. In Vitro Drug Release

**Figure 2:**
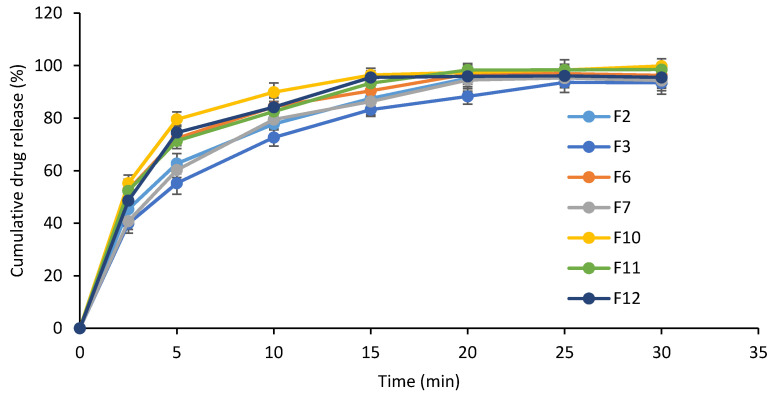
Cumulative in vitro release of DEX from in situ gels of different compositions.

The mean cumulative release percentages of DEX from the different in situ gel formulations were measured over 30 min (Figure 2). Formulations that are composed of Na alginate (F10, F11 and F12) and F6 that contains chitosan (0.5% *w*/*v*) showed the highest initial burst release of ca. 50% after 2.5 min. F3 and F7 that are composed of CP 934 (0.3% *w*/*v*) and chitosan (0.75% *w*/*v*), respectively, resulted in slower initial burst release (ca. 40%). After 30 min, almost complete release of the drug was observed from all formulations.

The kinetics of drug release from the different in situ gel formulations were studied by fitting the release data to various kinetic models. The determination coefficient (*r*^2^) values for the different models are presented in Table 2. The in vitro release data indicate that the release of DEX from in situ gels is best fitted to Korsemeyer–Peppas model, based on the highest correlation. Based on calculating the diffusion exponent of Peppas model, that was less than 0.5, Fickian diffusion mechanism is found to be dominant and is responsible for controlling the release of the drug from the different formulations [30,31].

Among the tested in situ gel formulations, F3 demonstrated enhanced mucoadhesive force, superior gelling capacity, reasonable pH and optimal rheological profile, and thus, it was selected for further pharmacokinetic and pharmacodynamic evaluations.

### 3.3. Stability Study

The stability study for the selected in situ gelling system (F3) was carried out at 4 °C, room temperature and at 40 °C for 30 d and 60 d to assess the long-term stability of the formulation as shown in Table 3. After 30 d and 60 d, no significant changes were observed in the various parameters studied (i.e., surface pH, viscosity, mucoadhesion and gelling capacity) either at room temperature, low temperature (i.e., 4 °C) or relatively high temperature (i.e., 40 °C).

### 3.4. Pharmacokinetics Analysis

**Figure 3:**
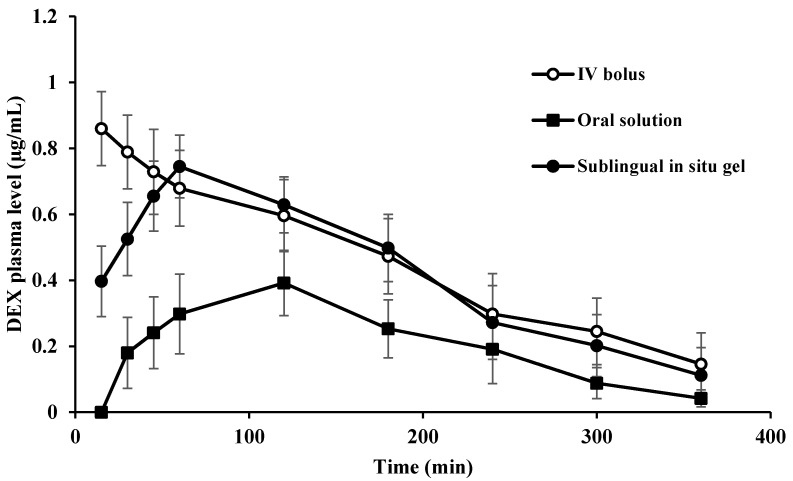
Plasma concentrations of DEX after IV, oral and sublingual administration.

F3 was selected for further pharmacokinetic evaluations due to its enhanced mucoadhesive force, superior gelling capacity, accepted pH and optimal rheological profile. The plasma levels of sublingually absorbed DEX from the in situ gel, F3, were assessed and compared to orally and IV administered DEX. Drug concentration in plasma was measured using a reported method previously described by our group [14]. DEX concentrations in plasma after sublingual, oral and IV administration are illustrated in Figure 3.

Fifteen minutes post-treatment, the sublingual in situ gel (F3) showed a detectable plasma concentration of 0.397 ± 0.107 µg/mL that increased progressively to reach the maximum concentration (C_max_) of 0.75 ± 0.05 µg/mL at T_max_ of 60 min, then decreased gradually and remained detectable for 6 h after sublingual administration (0.112 ± 0.08 µg/mL). IV administration of DEX provided a C_max_ of 0.86 ± 0.11 µg/mL followed by a progressive reduction to reach a plasma concentration of 0.15 ± 0.09 µg/mL after 6 h. At 30 min post-treatment, Oral DEX solution began to show a detectable plasma concentration of 0.18 ± 0.108 µg/mL, and the concentration remained detectable also for 6 h. The observed higher plasma concentrations achieved following sublingual and IV administration of DEX as compared to oral administration indicates that sublingual administration could bypass the first-pass metabolism that usually results in poor oral bioavailability of the drug (16%) [1].

The DEX pharmacokinetic data are summarized in Table 4. It could be noticed that sublingual administration of DEX in situ gel exhibited faster absorption rate in comparison to orally administered solution. This could be reflected by its shorter *T*_max_ value of 60 ± 11.3 min compared to 120 ± 9.5 min in the case of orally administered DEX. Additionally, *C*_max_ of DEX was markedly higher following sublingual administration as compared to oral administration (i.e., 0.75 ± 0.05 and 0.39 ± 0.05 µg/mL, respectively). This indicates higher rate of absorption upon sublingual administration.

Moreover, sublingual administration showed a significant increase in the area under the plasma concentration–time curve (AUC) (151.02 ± 17.27 µg min mL^−1^) as compared with that obtained upon oral administration of DEX solution (74.19 ± 14.43 µg min mL^−1^) (Table 4). The small AUC obtained after oral administration indicates the rapid clearance of the drug from the plasma due to its short half-life (53.61 ± 2.24 min) compared with that after sublingual in situ gel administration (87.12 ± 17.06 min). Worth noting is that sublingual administration of the selected DEX in situ gel achieved comparable pharmacokinetic results with that after IV administration of DEX solution. Moreover, the systemic bioavailability of DEX in the sublingual in situ gel was significantly higher than that obtained after oral administration (i.e., 89.22% vs. 43.83%, respectively), while showing no significant difference compared to IV administration.

### 3.5. Pharmacodynamic Studies

#### 3.5.1. Hot Plate Test

**Figure 4:**
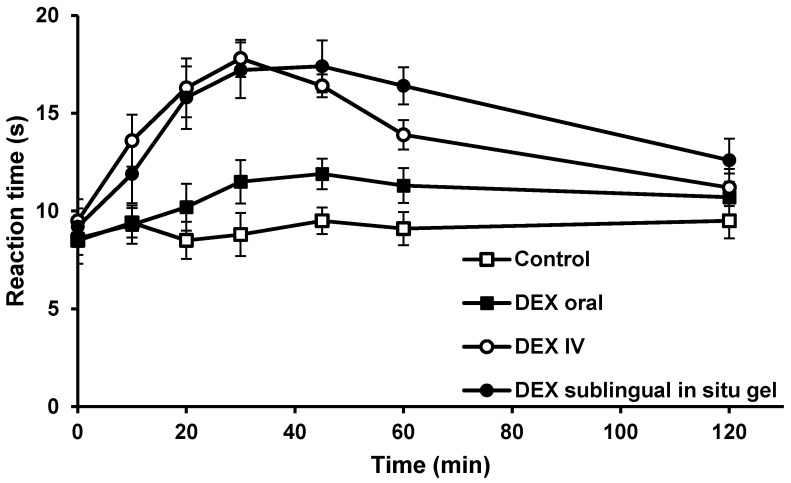
Reaction times in rats following sublingual administration of DEX in situ gel (F3), oral and IV administrations of DEX free drug solutions, as measured by the hot plate method.

Hot plate method was utilized to evaluate the reaction time for rats when placed on hot plates at specified time intervals upon the administration of the selected DEX in situ gel (F3) sublingually, compared to the orally and intravenously administered DEX solutions. Sublingual administration of F3 resulted in a significant increase in the reaction time for rats compared to DEX administered orally over 1 h, as shown in Figure 4. Interestingly, rats that received F3 showed no significant difference in the reaction time as compared to IV administration of DEX at all time intervals except at 60 min. At this time point (i.e., 60 min), F3 resulted in a significant increase in reaction time compared to results obtained after IV administration (i.e., from 13.9 ± 0.8 to 16.4 ± 1.0 s). This indicates the ability of DEX in situ gel to extend the duration of analgesia as compared to IV DEX. Details of the pharmacodynamic study results (i.e., hot plate method, measurements of systolic blood pressure and heart rate) are included in the Supplementary Materials (Tables S2–S4).

#### 3.5.2. Measurement of Systolic Blood Pressure

**Figure 5:**
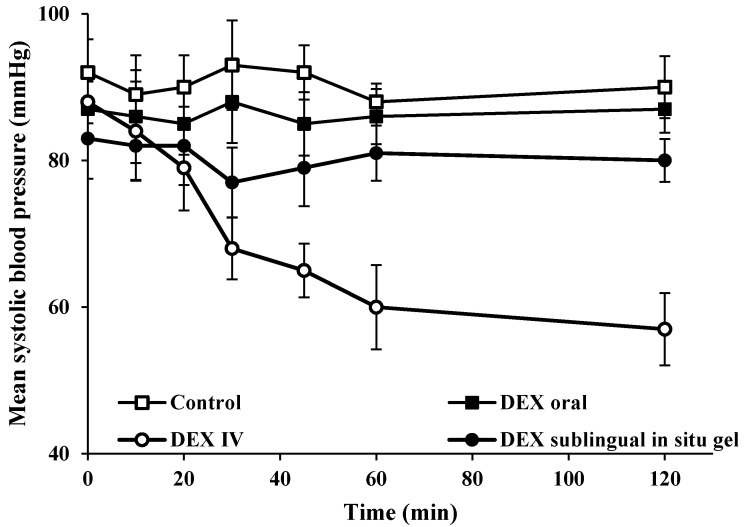
Effect of sublingual administration of DEX in situ gel (F3), oral and IV administration of DEX on the systolic blood pressure of rats.

Systolic blood pressure and heart rate were measured after oral, IV and sublingual administration of DEX to assess the associated sympatholytic effects of the drug. Mean systolic blood pressure measured in rats that received DEX intravenously was reduced significantly at 20 min compared to the measured blood pressure prior to treatment (i.e., from 88 ± 2.9 to 79 ± 5.8 mmHg, respectively) (Figure 5). After 20 min, the reduction in blood pressure in rats that received IV DEX was highly significant compared to the values obtained before treatment. This undesired effect (i.e., reduced blood pressure) after IV administration could be attributed to the binding of DEX to alpha-2 receptors located in the vasomotor centers in the brain stem, thus causing a reduction in sympathetic tone and consequently blood pressure [32]. Oral administration of DEX resulted in a non-significant difference in systolic blood pressure at all specified time points compared to measured values prior to the treatment. This could be ascribed to the extensive first pass metabolism of DEX after oral administration (16% oral bioavailability) [1] that requires higher doses to induce therapeutic effects. Sublingual administration of F3 resulted in no significant differences in the measured blood pressure before and after treatment. This provides evidence that sublingual administration of DEX resulted in similar pharmacologic effect (as shown in the hot plate method) as compared to IV administration, while overcoming the undesired effects associated with the latter. It has been shown that DEX is well-absorbed systemically through the oral mucosa, thus achieving a bioavailability of ca. 82% [33].

#### 3.5.3. Measurement of the Heart Rate

**Figure 6:**
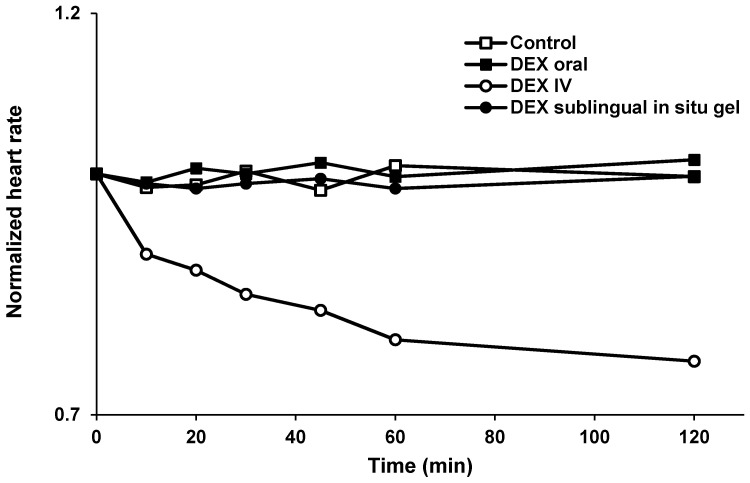
Normalized heart rates in rabbits following sublingual administration of DEX in situ gel (F3), oral and IV administrations of DEX free drug solutions.

Bradycardia is one of the undesired sympatholytic effects associated with the IV administration of DEX. To evaluate the effect of sublingual administration of DEX as an in situ gel, heart rates of rabbits after treatment with F3 was compared to that after oral and IV DEX administration. IV DEX resulted in a significant reduction in heart rate compared to the values observed prior to the treatment (Figure 6). These results are in an agreement with earlier studies that reported reduced heart rates upon IV administration of DEX [1,2,34]. On the contrary, sublingual administration of F3 showed no significant differences in the measured heart rates before and after treatment. Sublingual administration of DEX avoid the sudden increase in drug blood concentration as in the case of IV administration, thus preventing bradycardia, due to the long-lasting effects on parasympathetic efferent neurons [34].

## 4. Conclusions

In the current study, DEX in situ gels were developed and evaluated for their pH, gelling capacity, viscosity, general appearance, mucoadhesion and in vitro drug release. The optimized gelling system, F3, that demonstrated enhanced mucoadhesive force, superior gelling capacity, reasonable pH and optimal rheological profile was selected for further pharmacokinetic and pharmacodynamic evaluations. A significantly higher rate and extent of bioavailability was demonstrated after sublingual administration of the selected formulation compared to the oral administration of DEX. Comparable drug plasma levels were obtained upon sublingual and IV administration of DEX in situ gel and DEX solution, respectively. Sublingual administration of the selected gelling system (F3) demonstrated a sustained duration of analgesia in rats compared to the intravenously administered DEX solution, while maintaining normal systolic blood pressure and heart rate in the tested animals. Hence, sublingual administration of DEX in situ gel can be used as an alternative to IV administration of DEX solution for sedation during surgical operations to reduce anxiety, and to augment postoperative analgesia, while overcoming bradycardia and hypotension, the commonly reported side effects associated with IV administration of DEX.

## Figures and Tables

**Table 1:** pH, viscosity, mucoadhesive force and gelling capacity of the developed in situ gelling formulations.

Code	pH	Viscosity (Pa S) ^a^ (pH 6.8 ± 0.2, 37 °C)	Mucoadhesive Force (Pa)	Gelling Capacity ^b^
F2	4.9 ± 0.2	26.54 ± 2.84	3.90 ± 1.68	++
F3	5.1 ± 0.1	37.46 ± 0.82	4.83 ± 0.34	+++
F6	5.8 ± 0.1	23.74 ± 1.12	3.52 ± 2.51	+
F7	6.0 ± 0.1	32.66 ± 2.13	3.81 ± 1.41	++
F10	6.2 ± 0.3	17.50 ± 3.02	1.40 ± 1.33	+
F11	6.4 ± 0.2	19.53 ± 2.97	1.82 ± 1.74	+
F12	6.5 ± 0.2	24.56 ± 1.92	2.22 ± 2.24	++

Each point represents the mean ± SD (n = 3). ^a^ Solutions tested at 5 rpm. ^b^ + Gels after a few min, dissolves rapidly; ++, Immediate gelation, remains for few minutes; +++, Immediate gelation, remains for an extended period of time.

**Table 2:** Kinetics of DEX release from different in situ gel formulations according to different kinetic models.

Formulation	Determination Coefficient (*r*^2^)				(n) Korsmeyer–Peppas Equation
	Zero-Order	First-Order	Higuchi Diffusion	Peppas	
F2	0.8409	0.2895	0.9580	0.9796	0.30
F3	0.8729	0.15093	0.9739	0.9872	0.35
F6	0.7863	0.34022	0.9267	0.9621	0.24
F7	0.8455	0.2381	0.9595	0.9686	0.34
F10	0.7552	0.6593	0.9064	0.9308	0.22
F11	0.8032	0.5296	0.9369	0.971	0.25
F12	0.7757	0.2864	0.9193	0.9279	0.26

**Table 3:** Physicochemical evaluation of the most satisfactory in situ gel formulation, F3 during stability study (means ± S.D).

Time (d)	Zero Time			30 d			60 d		
Storage Temperature	4 °C	25 °C	40 °C	4 °C	25 °C	40 °C	4 °C	25 °C	40 °C
Surface pH *	4.9 ± 0.22	5.1 ± 0.19	5.0 ± 0.23	5.2 ± 0.20	4.9 ± 0.19	5.0 ± 0.22	5.1 ± 0.21	5.0 ± 0.15	5.1 ± 0.18
Viscosity (Pa S) (pH 6.8, 37 °C)	38.16 ± 0.91	37.46 ± 0.82	35.99 ± 0.88	38.44 ± 0.99	37.16 ± 0.78	35.76 ± 0.69	38.56 ± 0.72	36.21 ± 0.92	35.96 ± 0.96
Mucoadhesive force (Pa)	4.91 ± 0.29	4.83 ± 0.34	4.72 ± 0.44	4.89 ± 0.31	4.79 ± 0.54	4.76 ± 0.33	4.93 ± 0.24	4.81 ± 0.25	4.7 ± 0.62
Gelling Capacity	+++	+++	+++	+++	+++	+++	+++	+++	+++

+++, Immediate gelation, remains for an extended period of time. * Mean ± S.D (n = 3).

**Table 4:** Calculated pharmacokinetic parameters for DEX in plasma after sublingual administration of F3 compared to oral and IV of DEX.

Formulation	Pharmacokinetic Parameters							
	C_max_ (μg mL^−1^)	T_max_ (min)	V/F (min^−1^)	Cl/F (min)	K_el_ (min^−1^)	t_½_ (min)	AUC (μg min mL^−1^)	F (%)
DEX oral	0.39 ± 0.05	120 ± 9.5	1.02 ± 0.21	0.013 ± 0.00	0.0013 ± 0.00	53.61 ± 2.24	74.19 ± 14.43	43.83
DEX IV bolus	0.86 ± 0.06	-	0.89 ± 0.06	0.005 ± 0.00	0.007 ± 0.00	99.81 ± 15.2	169.26 ± 20.025 *	100 *
DEX sublingual (in situ gel F3)	0.75 ± 0.05	60 ± 11.3	0.76 ± 0.05	0.006 ± 0.00	0.008 ± 0.00	87.12 ± 17.06	151.02 ± 17.27 *	89.22 *

Data are represented as means ± SD (n = 5). * Significant differences compared to that of DEX oral. Abbreviations: DEX, dexmedetomidine; SD, standard deviation; C_max_, maximum concentration; T_max_, time of maximum concentration achieved after administration; V/F, apparent volume of distribution, Cl/F, apparent plasma clearance, Ka, absorption rate constant; t_½_a, absorption half-life; K_el_, elimination rate constant; t_½_, elimination half-life; AUC, area under the DEX plasma concentration–time curve; F, the absolute bioavailability.

## Data Availability

Not applicable.

## Supplementary Materials

The following supporting information can be downloaded at: https://www.mdpi.com/article/10.3390/pharmaceutics14020220/s1, Table S1: Physical state of various in situ gelling systems. CP 934 = Carbopol 934, HEC = Hydroxyl ethyl cellulose. HEC, BCK and boric acid are included in each formulation as 0.2, 0.01 and 0.5% *w*/*v*, respectively, and the final volume of each formulation is 100 mL. Table S2: Hot plate reaction times in rats following sublingual administration of DEX in situ gel, oral and intravenous (IV) administrations of DEX free drug solutions. Table S3: Effect of sublingual administration of DEX in situ gel, oral and intravenous (IV) administrations of DEX free drug solutions on the blood pressure in rats. Table S4: Heart rates in rabbits following sublingual administration of DEX in situ gel, oral and intravenous (IV) administrations of DEX free drug solutions.
