# Supplementary material for: Development of Sedative Dexmedetomidine Sublingual In Situ Gels: In Vitro and In Vivo Evaluations
Source: Pharmaceutics. 2022 Jan 18;14(2):220. doi: 10.3390/pharmaceutics14020220 (PMC8878141; doi:10.3390/pharmaceutics14020220)
Supplement: Supplementary file 1 [file pharmaceutics-14-00220-s001.zip › pharmaceutics-1542333-supplementary.pdf]

pharmaceutics

MDPI

Supplementary Materials: Development of Sedative Dexmedetomidine Sublingual In Situ Gels: In Vitro and In Vivo Evaluations

Ayat A. Allam, Nermin E. Eleraky, Nadeen H. Diab, Mahmoud Elsabahy Sahar A. Mohamed, Hala S. Abdel-Ghaffar, Nivin A. Hassan, Samia A. Shouman, Mervat M. Omran, Sahar B. Hassan and Noura G. Eissa

Table S1. Physical state of various *in situ* gelling systems. CP 934 = Carbopol 934, HEC = Hydroxyl ethyl cellulose. HEC, BCK and boric acid are included in each formulation as 0.2, 0.01 and 0.5% *w*/*v*, respectively, and the final volume of each formulation is 100 mL.

Code	CP 934	Chitosan	Na Alginate	HEC	Physical State at Non-Physiological Condition	Physical State at Physiological Condition (37 °C, pH 7.4)
	Concentration (% *w*/*v*)					
F1*	0.1	-------	-------	0.2	Clear transparent solution	Clear transparent viscous solution
F2	0.25	-------	-------	0.2	Clear transparent solution	Clear transparent gel
F3	0.3	-------	-------	0.2	Clear transparent solution	Clear transparent gel
F4*	0.5	-------	-------	0.2	Clear transparent gel	Clear transparent gel
F5*	-------	0.25	-------	0.2	Clear transparent solution	Clear transparent viscous solution
F6	-------	0.5	-------	0.2	Clear transparent solution	Clear transparent gel
F7	-------	0.75	-------	0.2	Clear transparent solution	Clear transparent gel
F8*	-------	1.0	-------	0.2	Clear transparent gel	Clear transparent gel
F9*	-------	-------	0.25	0.2	Clear transparent solution	Clear transparent viscous solution
F10	-------	-------	0.5	0.2	Clear transparent solution	Clear transparent gel
F11	-------	-------	0.75	0.2	Clear transparent solution	Clear transparent gel
F12	-------	-------	1.0	0.2	Clear transparent solution	Clear transparent gel

*excluded formulations due to their lack of flowing property at non-physiological conditions.

Table S2. Hot plate reaction times in rats following sublingual administration of DEX *in situ* gel, oral and intravenous (IV) administrations of DEX free drug solutions.

Groups	Reaction Time (s)						
	**Before treatment**	**10 min**	**20 min**	**30 min**	**45 min**	**60 min**	**120 min**
Control	8.5 ± 1.2	9.4 ± 0.8	8.5 ± 1.0	8.8 ± 1.1	9.5 ± 0.7	9.1 ± 0.9	9.5 ± 0.89
DEX oral	8.6 ± 0.9	9.3 ± 1.0	10.2 ± 1.2	11.5 ± 1.1	11.9 ± 0.8	11.3 ± 0.9	10.7 ± 1.22
DEX IV	9.5 ± 1.1	13.2 ± 1.3#	16.3 ± 1.5#	17.8 ± 1.0#	16.4 ± 0.6#	13.9 ± 0.8**	11.2 ± 0.95#
DEX sublingual (*in situ* gel F3)	9.2 ± 0.9	11.9 ± 1.5*	15.8 ± 1.6*	17.2 ± 1.4*	17.4 ± 1.3*	16.4 ± 1.0*	12.6 ± 1.1

Data are represented as means ± SD (n = 6). *Significant differences compared to that of the DEX oral solution ($p < 0.05$). #Non-significant differences compared to that of the DEX sublingual *in situ* gel. **Significant differences compared to that of the DEX sublingual *in situ* gel ($p < 0.01$).

Table S3. Effect of sublingual administration of DEX *in situ* gel, oral and intravenous (IV) administrations of DEX free drug solutions on the blood pressure in rats.

Groups	Mean systolic Blood Pressure						
	**Before treatment**	**10 min**	**20 min**	**30 min**	**45 min**	**60 min**	**120 min**
Control	92 ± 4.5	89 ± 5.3	90 ± 4.3	93 ± 6.1	92 ± 3.7	88 ± 2.5	90 ± 4.2
DEX oral	87 ± 3.8	86 ± 6.3#	85 ± 4.2#	88 ± 5.6#	85 ± 4.3#	86 ± 3.8#	87 ± 3.2#
DEX IV	88 ± 2.9	84 ± 6.8#	79 ± 5.8*	68 ± 4.2**	65 ± 3.7**	60 ± 5.8**	57 ± 4.9**
DEX sublingual (*in situ* gel F3)	83 ± 5.5	82 ± 4.7#	82 ± 5.3#	77 ± 4.8#	79 ± 5.2#	81 ± 3.8#	80 ± 2.9#

Data are represented as means ± SD (n = 6). #Non-significant differences compared to the measured blood pressure before treatment. *Significant differences compared to the measured blood pressure before treatment ($p < 0.05$). **Highly Significant differences compared to the measured blood pressure before treatment ($p < 0.01$).

Table S4. Heart rates in rabbits following sublingual administration of DEX *in situ* gel, oral and intravenous (IV) administrations of DEX free drug solutions.

Groups	Heart Rate						
	**Before treatment**	**10 min**	**20 min**	**30 min**	**45 min**	**60 min**	**120 min**
Control	294 ± 6.5	289 ± 7.2	290 ± 5.8	295 ± 4.9	288 ± 6.4	297 ± 5.8	293 ± 7.6
DEX oral	286 ± 4.8	283 ± 5.6#	288 ± 4.9#	286 ± 6.3#	290 ± 7.1#	285 ± 6.6#	291 ± 5.8#
DEX IV	300 ± 5.3	270 ± 6.4*	264 ± 4.8*	255 ± 7.3*	249 ± 6.6*	238 ± 4.8*	230 ± 3.9*
DEX sublingual (*in situ* gel F3)	328 ± 5.1	324 ± 4.7#	322 ± 5.3#	324 ± 3.8#	326 ± 3.9#	322 ± 5.2#	327 ± 6.2#

Data are represented as means ± SD (n = 5). #Non-significant differences compared to the measured blood pressure before treatment. *Highly significant differences compared to the measured blood pressure before treatment ($p < 0.01$).
